# Soybean Nodulation Response to Cropping Interval and Inoculation in European Cropping Systems

**DOI:** 10.3389/fpls.2021.638452

**Published:** 2021-06-04

**Authors:** Mosab Halwani, Moritz Reckling, Dilfuza Egamberdieva, Richard Ansong Omari, Sonoko D. Bellingrath-Kimura, Johann Bachinger, Ralf Bloch

**Affiliations:** ^1^Leibniz Centre for Agricultural Landscape Research (ZALF), Müncheberg, Germany; ^2^Department of Crop Production Ecology, Swedish University of Agricultural Sciences, Uppsala, Sweden; ^3^Faculty of Biology, National University of Uzbekistan, Tashkent, Uzbekistan; ^4^Faculty of Life Sciences, Humboldt-University of Berlin, Berlin, Germany; ^5^Faculty of Landscape Management and Nature Conservation, Eberswalde University for Sustainable Development, Eberswalde, Germany

**Keywords:** soybean, Bradyrhizobia, cropping interval, inoculation, nodulation, Central Europe

## Abstract

To support the adaption of soybean [*Glycine max* (L) Merrill] cultivation across Central Europe, the availability of compatible soybean nodulating Bradyrhizobia (SNB) is essential. Little is known about the symbiotic potential of indigenous SNB in Central Europe and the interaction with an SNB inoculum from commercial products. The objective of this study was to quantify the capacity of indigenous and inoculated SNB strains on the symbiotic performance of soybean in a pot experiment, using soils with and without soybean history. Under controlled conditions in a growth chamber, the study focused on two main factors: a soybean cropping interval (time since the last soybean cultivation; SCI) and inoculation with commercial Bradyrhizobia strains. Comparing the two types of soil, without soybean history and with 1–4 years SCI, we found out that plants grown in soil with soybean history and without inoculation had significantly more root nodules and higher nitrogen content in the plant tissue. These parameters, along with the leghemoglobin content, were found to be a variable among soils with 1–4 years SCI and did not show a trend over the years. Inoculation in soil without soybean history showed a significant increase in a nodulation rate, leghemoglobin content, and soybean tissue nitrogen concentration. The study found that response to inoculation varied significantly as per locations in soil with previous soybean cultivation history. An inoculated soybean grown on loamy sandy soils from the location Müncheberg had significantly more nodules as well as higher green tissue nitrogen concentration compared with non-inoculated plants. No significant improvement in a nodulation rate and tissue nitrogen concentration was observed for an inoculated soybean grown on loamy sandy soils from the location Fehrow. These results suggest that introduced SNB strains remained viable in the soil and were still symbiotically competent for up to 4 years after soybean cultivation. However, the symbiotic performance of the SNB remaining in the soils was not sufficient in all cases and makes inoculation with commercial products necessary. The SNB strains found in the soil of Central Europe could also be promising candidates for the development of inoculants and already represent a contribution to the successful cultivation of soybeans in Central Europe.

## Introduction

Soybean (*Glycine max* [L.] Merr.) is one of the most extensively cultivated crops worldwide, representing approximately 57 and 79% of the global pulse area and production in 2018, respectively (FAO, 2020). In Europe-28, however, areas under soybean cultivation are still below 1 million ha, and the region depends largely on imports from North and South America to meet its protein demand (Watson et al., 2017). The demand for soybeans from Europe is increasing, and there is a growing interest by farmers to experiment with the crop (Reckling et al., 2020) and to diversify their cropping systems that are dominated by cereals (Hufnagel et al., 2020). There are studies to identify potent indigenous and commercial Bradyrhizobia inoculants suitable for European conditions (Zimmer et al., 2016; Yuan et al., 2020) and attempt to incorporate soybeans in the no-till cover-crop system (Halwani et al., 2019). However, very little is known about the potential of soybeans in higher latitudes (Lamichhane et al., 2020; Schoving et al., 2020). Soybean seeds contain high nutrients for feed and food use, and the crop establishes nitrogen-fixing symbiosis with Bradyrhizobia, fixing 68% and 119 kg ha^−1^ of nitrogen aboveground from the atmosphere (Peoples et al., 2009). The Bradyrhizobia are either indigenous and present in the soils along with the appropriate hosts or are introduced with a new host by seed inoculation (Corman et al., 1987). In areas where soybeans were domesticated centuries ago, soybean-nodulating Bradyrhizobia (SNB) usually survive and are present in soils in high diversity, making the utilization of commercial inoculants less important (Zhang et al., 2011). In contrast, a soybean is a novel crop in Central Europe (Shurtleff and Aoyagi, 2016), and it has been shown that such soils lack native SNB (Crozat et al., 1987; Madrzak et al., 1995; Narożna et al., 2015). Thus, enhancing soybean expansion and productivity in Europe will also depend on effective inoculation, and farmers currently cultivate soybeans, using commercially available Bradyrhizobium inoculants (Zhang et al., 2003; Zimmer et al., 2016; Reckling et al., 2020).

Previous research in Central Europe has been mainly focused on the selection of the most efficient Bradyrhizobia strains from different inoculants under cold growing conditions (Kadiata et al., 2012) and their interaction with different early maturing soybean varieties (Zimmer et al., 2016; Kühling et al., 2018). In these studies, soybean inoculation with Bradyrhizobia increased the grain yield by up to 56%, protein content by 26% (Zimmer et al., 2016), chlorophyll content by 120% (Kühling et al., 2018); and improved nodulation and N_2_-fixation (Kadiata et al., 2012). These experiments tested the symbiotic performance of soybeans with inoculants in soils with no history of soybean cultivation. On the other hand, previously introduced strains, subsequently, might remain genetically isolated in soil or may undergo genetic changes to fit the local conditions (Perrineau et al., 2014). Indeed, inoculants are often applied to soils with soybean cultivation history and established SNB (Obaton et al., 2002; Narożna et al., 2015; Yuan et al., 2020). Little is known about the possible influences of the previously introduced SNB on the symbiotic performance of commercial inoculants. Research in the North and South America showed that the response of inoculation with commercial Bradyrhizobia inoculants differed in soils when soybean was included in previous rotations. This introduced SNB in the soil can either enhance, stay neutral or compete with the elite strain in the commercial inoculants (Thies et al., 1991; Piccinetti et al., 2013; Iturralde et al., 2019).

There is sufficient evidence regarding the presence of SNB in European soils at high latitudes with cold winters. The presence of indigenous bacteria can be seen as a chance for the SNB to adapt to European conditions. It may also cause a problem by escalating the competition between indigenous and inoculated strains. However, no information is available concerning the symbiotic performance of soybeans with the present SNB in the soil of Central Europe and their competition with commercial inoculants. The objectives of this study were to (i) investigate the nodulation and symbiotic efficiency of indigenous SNB in soils from different soybean cropping intervals, and (ii) assess the response of a soybean to commercial inoculants in the presence of indigenous SNB in a pot experiment.

## Materials and Methods

A pot experiment was conducted in growth chambers at the Leibniz Centre for Agricultural Landscape Research (ZALF), located in Müncheberg, Germany. Plants were grown from January to February 2018 for 40 days in tall and narrow 2.5-L plastic pots filled with 2.3-kg soil.

### Soil Sampling

Eleven soil samples with different soybean cultivation histories (Table 1) were collected from two locations in the northeast of Germany, Fehrow (N 51° 51′ 9.915″ E 14° 16′ 42.874″) and Müncheberg (N 52° 30′ 56.16″ E 14° 7′ 38.639″) in November 2017. At each location, soil samples were collected from five random spots at a depth of 0 to 20 cm. The main agronomic practices at both locations: The maize is amended with cattle manure in early spring; manure is immediately incorporated, using a chisel plow. Grass-clover mix is mowed 2–3 times per year, and the biomass is raked and baled. Weeds are managed using rotary hoeing and row cultivation until crop canopy closure.

**Table 1:** Crop sequence history in the sampling sites.

		Year of soil sampling	Pre-crops		
Location	SCI^1^	2017	2016	2015	2014
Fehrow	1	Soybean	Winter wheat	Grass-clover mix	Winter wheat
	2	Winter wheat	Soybean	Winter wheat	Grass-clover mix
	3	Grass-clover mix	Winter wheat	Soybean	Maize
	4	Maize	Grass-clover mix	Winter wheat	Soybean
	0	Grass-clover mix	Grass-clover mix	Grass-clover mix	Grass-clover mix
Müncheberg	1	Soybean	Winter wheat	Winter oilseed rape	Winter rye
	2	Oat	Soybean	Winter barley	Winter wheat
	3	Grass-alfalfa mix	Spring oat	Soybean	Maize
	4	Grass-clover mix	Grass-clover mix	Spring oat	Soybean
	0	Maize	Alfalfa-grass mix	Winter rye	Winter rye
	0^*^	Grass	Grass	Grass	Grass

1 Soybean cropping interval (year/s since the last soybean cultivation).

* No legumehistory at all.

Soybean cropping interval (SCI) refers to the year(s) since the last soybean cultivation. In the sampling sites with zero SCI, there is no history of soybean cultivation. In the other sampling sites, soybean cultivar “Merlin” had been cultivated, and seeds were inoculated with HiStick®, a commercial SNB strain from BASF. Similar agronomic practices were used in all sampled sites.

### Physical and Chemical Characteristics of Soil

Müncheberg soils consist of 65–77% sand, 17–27% silt, and 5–7% clay. Their particle size distribution categorizes the soil as slightly loamy sand. The field capacity lies between 9.5 and 12%. The soil in Fehrow is medium loamy sand with 8–12% sand, 10–40% silt, and 48–82% clay. Compared with Müncheberg soils, soils in Fehrow have a higher field capacity in the range of 10.5–21%. Soil pH in water ranged from neutral (pH 7.0) to slightly acidic (pH 6.0) in soil samples at both locations.

The carbon content in the soils of Müncheberg and Fehrow was 1–2% and <0.8%, respectively; while nitrogen contents were 0.1–0.2% and 0.05–0.08, respectively. At both locations, the contents of double lactate extractable phosphorus, exchangeable potassium, and magnesium ranged from 1.7–9, 2.9–12, and 0.9–1.8 g kg^−1^, respectively.

### Experimental Conditions

The seeds of early maturing soybean cultivar “Merlin” (maturity group 000) were surface sterilized by immersion in 10% v/v bleach NaOCl solution for 45 s and then in 70% ethanol for 45 s before being rinsed five times, using sterile water. Surface-sterilized, bold, and healthy seeds were sown two seeds per pot at a depth of 3 cm. There were two treatments: non-inoculation and inoculation with a commercial inoculant. The commercial soybean inoculant, HiStick®, obtained from BASF, North Carolina, NC, USA, containing *Bradyrhizobium japonicum* (4 × 10^9^ viable cells gram^−1^), was used for inoculation of soybean seeds. Each treatment was replicated six times.

A trap host approach, described by Howieson et al. (2016), was used to sow the surface-sterilized soybean seeds directly into the soil. The conditions in the growth chambers were a 16/8 h light regime and at a temperature range of 22/15°C of day/night, respectively (Martyniuk et al., 2016). The soil temperatures in the pot were measured, using a soil thermometer. The Soil–Plant Analysis Development (SPAD) was measured weekly, using chlorophyll meter SPAD 502 Plus (Konica Minolta Optics, Inc., Osaka, Japan).

### Plant Harvest for Assessing Nodulation and Plant Growth Parameters

After 40 days, the aboveground plant growth parameters, such as the height and dry weight of the plant shoots, were determined. Afterward, roots were washed carefully with water over a metal sieve to ensure minimum nodule loss and to reduce possible shedding and rupturing of nodules. The number of nodules and average weight of nodules per plant were determined. Nitrogen concentrations in the plant green tissues were measured according to the Kjeldahl method. Olympus AT200 auto analyzer was used to measure the nitrogen contents in shoots.

#### Leghemoglobin Test

Leghemoglobin in the soybean nodules was determined by using the modified method of Wilson and Reisenauer (1963). The leghemoglobin was extracted with Drabkin’s solution and measured colorimetrically as the CMLHb complex after centrifuging. The leghemoglobin content (mg g^−1^) of each sample was determined directly from the calibration curve.

#### Root Architecture

Each plant root system was washed and cleansed of soil particles. Harvested roots were scanned in water with a flatbed scanner (Epson Expression 10,000 xL, SEIKO Epson CORPORATION, Japan, resolution 2,400 dpi). Root volume, root length, a root surface area, a root diameter, and root tips were measured from the scanned images using the commercial root-scanning system WinRhizoᵀᴹ 2007 (Régent Instruments Inc., Canada).

#### Statistical Analysis

To evaluate the response of nodulation and symbiotic performance of SNB to a soybean cropping interval, separate data of each location from non-inoculated treatments were subjected to the variance analysis. The differences between the means were tested by applying Tukey’s test. To evaluate the response of nodulation and symbiotic performance to inoculation, data collected from each location were divided into sites with and without soybean history. After satisfying the assumptions for normality and homogeneity of variance, the software SAS® 9.2 (SAS Institute Inc., Cary, NC, United States) was employed, using the PROC MIXED procedure for data evaluation. The Pearson’s correlation coefficients among the nodule, plant growth parameters, and root architecture traits were calculated, using the JMP Pro® (version14.3) software for multivariate data analysis (SAS Institute Inc., Cary, NC, United States). The results were expressed at the *p* < 0.05 (^∗^) and *p* < 0.01 (^∗∗^) levels of probability, respectively.

## Results

### Plant Growth Response to Soybean Cropping Interval Without Inoculation

The analysis of variance showed that the plant height, dry weight of shoot, and SPAD-value were not significantly (*p* ≤ 0.05) affected by SCI at both locations. The plant heights ranged from 22- to 27-cm plant^−1^ and from 25- to 27-cm plant^−1^, while the weights ranged from 1.2- to 1.6-g plant^−1^ and from 1.3- to 1.4-g plant^−1^, and its SPAD-values ranged from 35 to 36 and from 29 to 32 in Fehrow and Müncheberg, respectively. Meanwhile, the root system architecture traits divided the soil samples in Fehrow into two groups. The first group consists of soils with 2-year SCI and soils with 4-year SCI. The second group consists of soils with no soybean history, soils with 1-year SCI, and soils with 3-year SCI, which were statistically the same in all measured root system architecture traits. Root length ranged from 17 × 10^6^ cm to 20 × 10^6^ cm and from 22 × 10^6^ cm to 25 × 10^6^ cm, a surface area ranged from 27 × 10^5^ cm^2^ to 34 × 10^5^ cm^2^ and 36–45 × 10^5^ cm^2^, a root volume ranged from 33 × 10^2^ cm^3^ to 44 × 10^2^ cm^3^ and from 47 × 10^2^ cm^3^ to 66 × 10^2^ cm^3^, a root diameter ranged from 0.50 to 51 mm and from 0.52 to 0.58 mm, and root tips ranged from 19 × 10^2^ to 25 × 10^2^ and from 33 × 10^2^ to 40 × 10^2^ in the first and second groups, respectively. Plants grown in Müncheberg soils with no soybean or legume history had root length of 27–29 × 10^6^ cm, a root surface area of 46–62 × 10^5^ cm^2^, a root volume of 64–106 × 10^2^ cm^3^, a root diameter of 0.6–0.68 mm, and root tips of 35–53 × 10^2^. These root system architecture traits were significantly lower in all soils with 1–4 SCI and ranged as follows: root length from 20 × 10^6^ to 24 × 10^6^ cm, a root surface area from 37 × 10^5^ cm^2^ to 42 × 10^5^ cm^2^, root volume from 54 × 10^2^ cm^3^ to 60 × 10^2^ cm^3^, a root diameter from 0.57 to 0.59 mm, and root tips from 27 × 10^2^ to 34 × 10^2^.

### Nodulation Response to a Soybean Cropping Interval Without Inoculation

Results of the nodulation rate, the average weight of nodules, leghemoglobin content, and nitrogen concentration in the green tissue were significantly different between locations and were analyzed separately.

#### Nodulation Response at the Location Fehrow

The nodule number formed on the roots of soybean plants was significantly affected (*p* = 0.0036) by the SCI at the location Fehrow (Table 2). The nodule number formed on plants grown in soils with 1- and 2-year SCI was not significantly different from soils with 3- and 4-year SCI, and soils with no soybean history. However, plants grown in soils with 3-year SCI formed significantly more nodules than that with 4-year SCI and no soybean history. The average weight of nodules was significantly (*p* = 0.0095) higher in plants grown in all soils with no soybean history compared with plants grown in soil with 1–4 SCI. The average weight of nodules was 13.1 mg for soil samples with no soybean history, while, in soils with 1–4 SCI, the average weight of nodules ranged from 3.9 to 5.1 mg (Table 2).

**Table 2:** Influence of soybean cropping interval on nodulation rate, average weight of nodules, content of leghemoglobin in nodules as well as nitrogen concentration in plant shoot at Fehrow.

SCI^1^	Nodulation rate (nodule plant^−1^)	Average weight of nodule (mg)	Leghemoglobin content (mg g^−1^)	Nitrogen concentration in plant (%)
1	63^ab^	5.1^b^	24.2^a^	3.2^c^
2	46^ab^	3.9^b^	15.6^bc^	3.2^d^
3	83.7^a^	4.4^b^	23.4^ab^	3.7^a^
4	41.7^b^	4.2^b^	10.3^c^	3.5^b^
0	40^b^	13.1^a^	18.6^ab^	3.1^e^

Data was collected from non-inoculated plants. Means followed by a common letter are not significantly different by the Tukey’s test at the 5% level of significance.

1 Soybean cropping interval (year/s since the last soybean cultivation).

Among the soil samples with 1–4-year SCI, the leghemoglobin contents of nodules were affected significantly (*p* = 0.0003) by SCI. However, the leghemoglobin content of nodules in soil with no soybean history was 18.6 mg g^−1^ and was similar to values obtained in sites with 1–4 SCI. Nitrogen concentration in the plant tissue was significantly (*p* < 0.0001) higher in plants grown in all soils with 1–4 SCI than the plants grown in soil with no soybean history (Table 2).

#### Nodulation Response at the Location Müncheberg

The plants grown in Müncheberg soil with no soybean or no legume history formed significantly (*p* = 0.001) the lowest number of nodules of 7 and 3 nodule plants^−1^, respectively (Table 3). The number of nodules did not differ among soils with no soybean history, 1-year SCI and 3-year SCI. Plants grown in soil with 2-year SCI formed the highest number of nodules of 41 plants^−1^ and were similar to plants grown in soil with 4-year SCI (Table 3). The average weight of nodules was significantly (*p* =0.045) higher in plants grown in all soils with no soybean history compared with the plants grown in soil with 1–4 SCI. The average weight of nodules ranged from 44.4 to 50 mg for soil samples with no soybean history, while it ranged from 9.1 to 24.6 mg in the soils with 1–4 SCI (Table 3). The highest leghemoglobin content of 46 mg g^−1^ of fresh nodule compared with all other soils. Among the soils with soybean history, the leghemoglobin contents ranged from 16 to 30 mg g^−1^ with no statistical differences between soils with 1, 2, and 4 SCI.

**Table 3:** Influence of soybean cropping interval on nodulation rate, average weight of nodules, content of leghemoglobin in nodules as well as nitrogen concentration in plant shoot at Müncheberg.

SCI^1^	Nodulation rate (nodule plant^−1^)	Average weight of nodule (mg)	Leghemoglobin content (mg g^−1^)	Nitrogen concentration in plant (%)
1	10^cd^	24.6^b^	28.7^bc^	2.4^e^
2	40.7^a^	13.2^b^	30.2^b^	3.1^b^
3	22.7^bc^	9.1^b^	15.7^c^	2.9^c^
4	28^ab^	14.5^b^	17.6^bc^	3.2^a^
0	7.3^cd^	44.4^a^	29^bc^	2.7^d^
0^*^	2.7^d^	50^a^	45.9^a^	2.1^f^

Data was collected from non-inoculated plants. Means followed by a common letter are not significantly different by the Tukey’s test at the 5% level of significance.

1 Soybean cropping interval (year/s since the last soybean cultivation).

* No legume history at all.

Nitrogen concentration in the plant tissue was significantly (*p* = 0.0004) higher in soybean plants grown in soil with 1–4 year SCI compared with that grown in soil with no legume history (Table 3). Tissue nitrogen content in soil with no legume history was 2.1%, while it ranged from 2.4 to 3.2% for soils with 1–4 SCI.

### Nodulation and Plant Growth Response to Inoculation and Soybean Cropping Intervals

**Figure 1:**
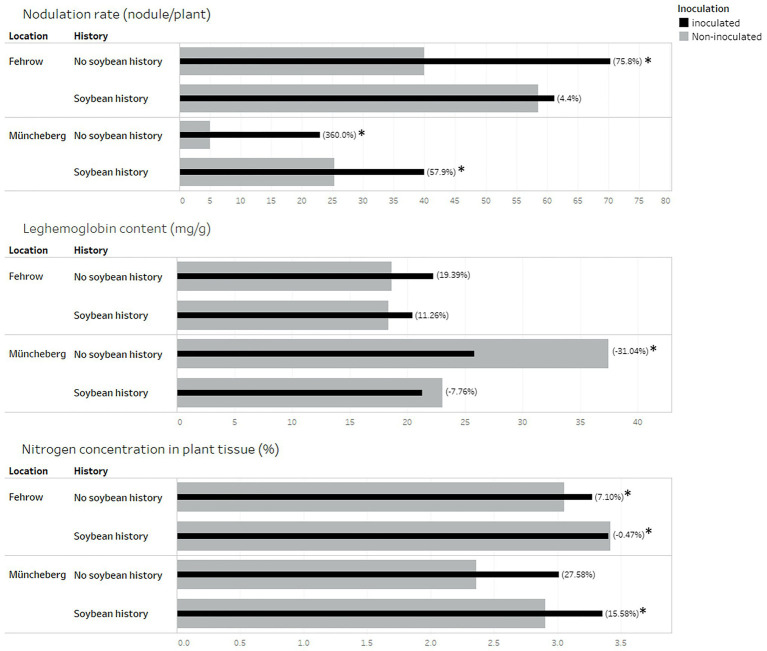
Influence of inoculation on nodulation rate, content of leghemoglobin in nodules as well as nitrogen concentration in plant shoot. Soil samples were collected from two locations Fehrow and Müncheberg in Germany. Soybean was introduced in the soil with soybean history since 1–4 years or never introduced in the soil with no soybean history. The black bars represent the inoculated plant. Gray bars represent the non-inoculated plants. Stars (*) indicate a significantly different between inoculated and non-inoculated plants by the student’s *t*-test at the 5% level of significance. Numbers in brackets indicate the percentage difference mean, either higher or lower as shown, between inoculated and non-inoculated plants.

Inoculation showed a significant influence on the nodulation rate in the soil with no soybean history. Relative to non-inoculation, the nodulation rate increased by 75.8% (*p* = 0.017) and 360% (*p* = 0.0041) with commercial inoculant in Fehrow and Müncheberg soils with no soybean history, respectively (Figure 1). In the soils with soybean history, the response to inoculation differed between the locations. While no significant (*p* = 0.82) differences were found between inoculated and non-inoculated plants grown in Fehrow soils, there was a significant increase (*p* = 0.049) in the nodulation rate by an average of 57.9% in Müncheberg soils with soybean history (Figure 1).

Inoculation showed a significant (*p* < 0.0001) influence on the average weight of nodules in the soil with no soybean history at both locations. In Fehrow, the average weight of nodules reduced from 13.1 to 7.2 mg, following inoculation. Also, the average weight of nodules reduced from 47.2 to 10.5 mg with inoculation in Müncheberg. In contrast, the average weight of nodules did not differ significantly (*p* = 0.34 and *p* = 0.133) with inoculation in the soil samples with soybean history in Fehrow and Müncheberg, respectively.

The leghemoglobin contents of soybean nodules did not increase significantly by inoculation. Instead, leghemoglobin content decreased significantly (*p* = 0.046) by 31% with inoculation at Müncheberg soil with no soybean history (Figure 1).

The shoot nitrogen concentration showed a positive response to the commercial inoculant tested in the soils with no soybean history at both locations, i.e., Fehrow (*p* = 0.004) and Müncheberg (*p* = 0.0034). Soybean tissue nitrogen concentration increased in inoculated soils with no soybean history by 7.1% at Fehrow and 27.6% at Müncheberg. In soils previously cultivated with soybeans, the inoculated plants had a significantly (*p* = 0.0004) higher tissue nitrogen concentration of 15.6% compared with the non-inoculated plant at Müncheberg (Figure 1). However, the plant tissue nitrogen concentration did not increase significantly (*p* = 0.8185) with inoculation in the soils with soybean history at Fehrow. The plant height, dry weight of shoot, and SPAD value as well as all root architecture traits did not show a positive response to the commercial inoculant tested in this experiment (data not presented).

The correlation matrix of the studied parameters revealed positive correlations between the nodulation rate and chlorophyll content measured by SPAD (*r*^2^ = 0.69; *p* < 0.05) and nitrogen concentration in the plant tissue (*r*^2^ = 0.90; *p* < 0.001). Furthermore, the correlation analysis also showed a significant (*r*^2^ = 0.84; *p* < 0.01) positive relationship between SPAD values measured and nitrogen concentration in the green tissue. In contrast, the dry weight of the shoot showed a significantly positive correlation with all root architecture traits (*r*^2^ from 0.46 to 0.52; *p* < 001). A significant positive relationship existed among the root architecture traits: root length, a surface area, volume, a diameter, and tips (*r*^2^ ranged from 0.69 to 0.96; *p* < 0.01). No correlations were found for the nodulation rate and the following parameters: leghemoglobin content in the nodules, plant height, dry weight of shoot, and all root architecture traits.

## Discussion

This study confirmed the presence of indigenous SNB on the soybean plant grown at both locations (Fehrow and Müncheberg) with no soybean history, as well as with no legume history in Müncheberg. The observed nodules had a very high leghemoglobin content (Tables 2 and 3), which suggests evidence of symbiotic nitrogen fixation (Ott et al., 2005). A minimal number of nodules observed even in the first soybean cultivation at non-inoculated conditions were already reported in Germany (Kühling et al., 2017). It might be related to the ability of soybeans to establish symbiosis with other rhizobia, which has a broad range of host plants, confirming the results obtained in northeastern China (Yan et al., 2014). Yan et al. (2014) isolated a high diversity of soybean Bradyrhizobia from grassland, which has been cultivated for 28 years with a mixture of legume and non-legume wild species. Yuan et al. (2020) selected one symbiotic isolate, GMM49, which is closely related to *R. tropici* and *R. lusitanum*, from the soil with no legume history in Germany. This isolate is capable of forming nodules with soybeans more effectively than the commercial isolates. Another possibility might be that the soil was contaminated by Bradyrhizobia through wind and/or water erosion as well as through farming equipment used over the years (Larson, 2013; Mason et al., 2016). Vargas et al. (1994), in Brazil, found a strain of *B. japonicum* in soil samples, although the sampled site was thousands of kilometers away from the area where it had been introduced as an inoculant. However, at both locations in the present study, the nodulation rate of plants grown in soils with no soybean or legume history was lower than plants grown in soils with 1–4 years SCI and was accompanied by a high average nodule weight per plant and low shoot nitrogen concentration. These observations support the assertion that SNB can be present in soils at low population densities (Thies et al., 1991).

The nodulation rate was high when the inoculated soybean seeds were grown in the soils from the sites with no soybean history, which was initially devoid of SNB or had it in low populations (Figure 1). The positive response to inoculation is consistent with the other experiments conducted under controlled conditions in growth chambers (Kadiata et al., 2012) and natural soil conditions (Piccinetti et al., 2013; Zimmer et al., 2016; Kühling et al., 2017; Reckling et al., 2020). The higher nodulation rate was associated with a higher nitrogen concentration in the plant shoots (Figure 1), which was also found in other studies (Koutroubas et al., 1998; Sogut, 2006; Kadiata et al., 2012; Kühling et al., 2018). These observations indicate that the native or nonspecific soybean strains present in the soil are neutral and did not compete with the commercial inoculants (Sinclair and Nogueira, 2018). However, the leghemoglobin content of the nodules formed from these native or nonspecific soybean strains was as high as in the elite strains applied (Figure 1), which indicates good coordination between SNB and a plant host (Ott et al., 2005) and implies that their establishment in the soil at low population density was the main reason for their low nodulation rates (Thies et al., 1991). Increasing the nodulation rate by inoculation corresponded with decreasing an average nodule weight per plant and leghemoglobin content as well as increasing nitrogen concentration in plant tissues. This observation reflects a high nitrogenase enzyme activity in the small nodules despite the low leghemoglobin contents. Singh and Varma (2017) indicated that the presence of leghemoglobin is essential for the nitrogenase enzyme, but vice versa is true, and the relationship between leghemoglobin content and nitrogenase activity varies, depending on the strain, plant host, nodule size, and nodule age.

The non-inoculated soybean grown in soils with 1–4-year SCI formed more nodules than those grown in soil with no soybean history (Tables 2 and 3). These established SNBs were active and had sufficient leghemoglobin content that played an essential role in the N_2_ fixation of soybean nodules by facilitating O_2_ supply to the Bradyrhizobia for respiration (Ohyama et al., 2011) and, at the same time, protected the nitrogenase enzyme from oxygen denaturation (Singh and Varma, 2017). As a result, the shoot nitrogen concentration of soybean plants grown in soils with 1–4-year SCI was higher than those grown in soils with no soybean history. This finding suggests that the inoculated strains had been originally established in these soils or had changed genetically to fit the local conditions (Melchiorre et al., 2011; Perrineau et al., 2014). According to Nazir et al. (2013), introduced SNB strains could either become well adapted to the soil environment, displace a native population, and might occupy its niche (Nazir et al., 2013). This confirms the results of Yuan et al. (2020), who showed the close genetic relationship between some Bradyrhizobium isolates from soils in Germany and elite strains from commercial inoculants.

The nodulation rate and leghemoglobin content in the nodules, as well as plant tissue nitrogen concentration response to varying SCI, were significantly different between non-inoculated plants grown in soils with 1–4 year SCI history at both locations. However, these parameters did not show a consistent trend among the different SCI (Tables 2 and 3). This variation in symbiotic performance reflects the low effects of the introduced SNB strains in the soil or its nodulation activities by SCI. Factors such as soil management, crop rotation, organic fertilizer use (Triplett and Sadowsky, 1992) and land use patterns (Yan et al., 2014), as well as soil characteristics (Revellin et al., 1996), are likely to contribute to the variability in the response.

We observed significant variability between the locations in response to inoculation in the soils from sites with soybean history but were not able to identify the reasons for these differences (soils, climate, and management were different). A comparative analysis of the soybean rhizobia symbiotic genes in Germany showed no significant differences in *nodD* and *nifH* genes, which indicates that the soybean rhizobia symbiotic genes in Germany belong to only one type (Yuan et al., 2020). Moreover, in multilocus sequence analysis (MLSA), the majority of isolates were identified as *Bradyrhizobium*, and some isolates were shown to belong to the genus *Rhizobium*. The isolates identified as *Rhizobium* did not have the ability to form nodules on soybeans, depending on the phylogenetic analysis of symbiotic genes for *nodD* and *nifH* (Yuan et al., 2020).

In Fehrow, the nodulation rate, leghemoglobin, content and shoot nitrogen concentration in soil with soybean history were similar for non-inoculated and inoculated soybean seeds, showing no response to the commercial inoculation (Figure 1). This finding is in consonance with several previous studies in France (Revellin et al., 1996; Obaton et al., 2002) and at 73 environments in the USA (De Bruin et al., 2010; Mason et al., 2016). It is, however, in contrast to a common perception that the nodulation rate and symbiotic performance improve by using the inoculants containing elite strains of Bradyrhizobia (Mendes et al., 2004; Argaw, 2014). With respect to the results of Thies et al. (1991) and Mendes et al. (2004), who indicated that the response of legumes to rhizobial inoculants was inversely related to the population of present rhizobia in the soil, the modest response to commercial inoculant refers to the large populations of introduced SNB present in the soil and gives rise to two assumptions. Similar observations were reported for soils in Poland, where Bradyrhizobia strains were re-isolated 17 years later and compared with the same strains from the original inoculum strains. The strains remained viable, symbiotically capable, and had equal numbers, although a soybean was never grown in the field (Narożna et al., 2015).

On the other hand, there was a positive response of inoculation on a nodulation rate and shoot nitrogen concentration in soils from sites with soybean history in Müncheberg (Figure 1), representing a reduction in the introduced SNB abundance in the soil (Sinclair and Nogueira, 2018). The neutral response of leghemoglobin to inoculation refers to the high ability of established SNB to form a symbiotic association with a plant host (Ott et al., 2005). The decline in numbers of established SNB is likely to be a response to the direct and indirect harsh environmental conditions attributable to the sandy soil and drought conditions in the sampled area (Sinclair and Nogueira, 2018). Dudeja and Khurana (1989) reported degrees of the abundance of the Bradyrhizobia sp. (Cujanur) CC 1021 strain after 90 days of inoculation in the sandy loam soil for 2 years.

Inoculation with commercial inoculant should enhance growth parameters (García et al., 2004) as well as root system architecture traits (Yang et al., 2017). Nonetheless, our results showed no increase and even a slight decrease in SPAD value, plant height, and root architecture traits with inoculation (data not presented). This result is possibly due to the substantial demand for carbohydrates and nutrients for symbiotic microorganisms like Bradyrhizobia. The photosynthetically derived carbon from the legume host plants during the symbiosis process may constitute a possible competitive effect during the nodulation and nitrogen fixation (Thies et al., 1991; Reich et al., 2006; Aleman et al., 2010; Li et al., 2016). Takahashi et al. (1995) and Guo et al. (2011) indicated that soybean roots were shorter, and the root surface was limited by inoculation of the seed with symbiotic rhizobia.

## Conclusion

Nodulation and shoot nitrogen concentration differed among the varying SCIs in non-inoculated soils, but there was no consistency in the trend in both locations. A significant variability between the locations in response to inoculation with commercial inoculants was observed in the soils with soybean history. While a nodulation rate, leghemoglobin content, and shoot nitrogen concentration were similar to non-inoculated and inoculated soybean plants at the location Fehrow, a positive response to inoculation was observed at the location Müncheberg. Furthermore, the results of this study emphasize the presence of viable and symbiotically competent SNB strains in soils previously cultivated with soybeans. These previously introduced and adapted SNB whose presence in soil influenced the symbiotic performance of the commercial inoculants are promising candidates for ensuring effective inoculation in cold conditions of Central Europe. Further investigations, especially on the adapted SNB strains that fit the environments in Central Europe, are urgently needed.

## Data Availability Statement

The raw data supporting the conclusions of this article will be made available by the authors, without undue reservation.

## Author Contributions

MH, RB, and DE conceived and designed the experiment. MH performed the agronomic analysis and wrote the manuscript. MR, JB, DE, RO, and SB-K provided many helpful conceptual discussions. All authors contributed to the article and approved the submitted version.

### Conflict of Interest

The authors declare that the research was conducted in the absence of any commercial or financial relationships that could be construed as a potential conflict of interest.
